# Foreign nurse educators’ lived experiences of incivility: The case for Botswana

**DOI:** 10.4102/curationis.v43i1.2162

**Published:** 2020-12-21

**Authors:** Gloria B. Thupayagale-Tshweneagae, William M. Baratedi, Botshelo R. Sebola, Samuel Raditloko, Kefalotse S. Dithole

**Affiliations:** 1 Department of Health Studies, School of Social Sciences, University of South Africa, Pretoria, South Africa; 2 School of Nursing, Faculty of Health Sciences, University of Botswana, Gaborone, Botswana; 3 School of Public Health, Faculty of Health Sciences, University of Botswana, Gaborone, Botswana

**Keywords:** Botswana, discrimination, experiences, foreign nurse educators, hostile behaviours

## Abstract

**Background:**

In light of current economic prosperity and subsequent attainment of upper-middle-income country status, Botswana attracted nurse educators from other African countries. Within this cross-cultural environment, anti-immigrant sentiments have catalysed incidents of incivility, affecting the quality of teaching and learning outcomes.

**Objectives:**

The aim of this study was to explore experiences of incivility amongst foreign nurse educators and how it impacts their work and livelihood.

**Method:**

This study employed a qualitative approach, using interpretive phenomenology. In-depth interviews using a developed guide were conducted with 13 foreign nurse educators working as nurse educators in Botswana. Thematic analysis was conducted in accordance with interpretive phenomenology, where transcriptions were drafted after each interview.

**Results:**

Three themes emerged from the study findings: hostile behaviour, discrimination and inequitable application of procedures and processes. Discrimination as a theme has two sub-themes, namely, workplace injustice and individual injustice.

**Conclusion:**

The study found that foreign nurse educators working in Botswana experience incivility. From the findings, the study strongly recommends application of equitable job opportunities to all employees, including foreign nurse educators who are employed to meet the shortage of nurse educators in Botswana. The researchers are of the opinion that the nurse educator shortage will persist in Botswana, partly because of the nursing profession rendered unattractive by hostile social interactions amongst nurse educators’ exclusion of foreign nurses from benefits enjoyed by local nurse educators, workplace injustice targeting foreign nurse educators, as well as inequitable application of policies and processes that affect foreign nurse educators.

## Introduction

Incivility in nursing education has been defined as group of behaviours that are disruptive, such as being rude to each other, name-calling, withholding important information, gossip and belittling others (Muliira et al. 2017). Researchers (Spiri, Brantley & McGuire 2017; Williamson 2018) have further stated that a lack of respect, the use of derogatory statements and uncaring behaviour are other forms of incivility amongst nurse educators that have a negative impact on the teaching and learning process. Palumbo (2018) describes incivility amongst nurse educators as unfortunate as it disrupts the overall teaching and learning of nursing students and demoralises faculty members.

The Government of Botswana recognises the importance of having skilled manpower for delivering its policies and protection of its citizens. Through the Ministry of Health and Welfare, the government purports to create an appropriately skilled, highly motivated, client-focused health workforce. The ministry has embraced the role of training health personnel who will in turn assist in caring for its people. Five government colleges and two colleges owned by churches and fully funded by the government have been built and are offering nursing education. However, the shortage of trained and qualified staff remains a challenge within the same institutions. The institutions, in their quest to expand to meet the demands of the country and be competitive with other private and foreign institutions, are confronted with over-stretching the workforce. The ministry, therefore, came up with a National Policy that encompassed recruitment of expatriates to fill scarce positions, particularly those that are technical in nature (Ministry of Health 2011).

Botswana has been cited as a country of good governance since its independence in 1966. Because of the country’s administrative prudence, the country has also been known to be tolerant and accepting of foreign nationals based on the large-scale import of skilled personnel. However, the attitude of acceptance has changed over time. According to Campbell and Oucho (2003), the change in attitude is borne out of the belief that foreign nationals bring practices that disrupt the social fabric of Botswana. Fundamentally, the growth of intolerance seems related to actual changes in migration patterns to the country. For example, the economic and political situation in neighbouring Zimbabwe has resulted in a significant increase of immigrants. Furthermore, the media and the politicians have also worsened the negative awareness of immigrants and identified it as a problem. As a result, the people also view it in the same manner.

A shortage of nurse educators in various countries such as Botswana, Lesotho, Swaziland and South Africa has led to the recruitment of foreign nationals who come to host countries for presumed better prospects. However, the majority of them are faced with xenophobic antipathy from local nursing co-workers, and incivility in their work (Williamson 2018). In light of relative economic prosperity and subsequent attainment of upper-middle-income country status, Botswana has attracted nurse educators from various African countries. Within this cross-cultural environment, anti-immigrant sentiments have catalysed incidents of incivility amongst nurse educators, with adverse effects on both the quality of the teaching and learning outcomes.

Campbell and Oucho (2003) is of the view that there is insufficient evidence to conclude that Botswana’s employees do not like foreigners, but rather the intolerance appears to emanate from material and cultural factors to protect job opportunities for Botswana. Their view is not conclusive, as recent research (Spiri et al. 2017; Williamson 2018; Wright & Hill 2014) reported escalating episodes of incivility amongst nurse educators. It is within this background that the researchers undertook this study.

## Methods

### Design

A qualitative approach using interpretive phenomenology was used in this study to explore the lived experiences of foreign nurse educators working in Botswana (Creswell 2009). Qualitative research helps to explore and understand meanings individuals and groups attribute to a social and human problem (Williamson 2018). Phenomenology explores in detail the process of understanding the experiences of individuals and how they interpret them (Smith & Ostborn 2015).

### Setting and sampling

This study was conducted at three nursing colleges in Botswana that employ foreign nurse educators. The three sites were in Francistown, with six foreign nurse educators; Gaborone, with five nurse educators; and Kanye, with two foreign nurse educators. The first two institutions are government owned and the last institution is supported by the Government of Botswana, but is administrated by the Seventh Adventist Church.

### Data collection

Interviews were conducted following a self-developed guide. These were conducted between July and September 2019. The interviews lasted for 45–90 min and were audio-taped by the first author with the participants’ approval. The first author is a citizen of Botswana, who has been working in South Africa for the past 10 years. However, during data collection, the interviewer acknowledged her values on being a foreign nurse educator and used this awareness not to be prejudiced in any way. The third author took notes during the interviews. All the interviews started with one opening statement: ‘tell me your experiences of working as a nurse educator in Botswana’. Depending on the answer, this statement was followed by probing questions such as ‘how has this impacted your job?’ or ‘did you know that this would happen to you?’

All the participants were given an information leaflet and a consent form to sign before commencement of the interview. Interviews were conducted in the participants’ offices, which allowed for privacy.

### Data analysis

Data analysis began concurrently with data collection. Each transcript was drafted and read directly after the interview. Two authors read the transcripts several times. After the analysis was completed, and meanings and themes emerged, a sample was sent to five randomly selected participants to verify the findings. For member checking, each of the five participants was sent their own data because codes were used for their identification. Only one participant refuted the theme that was aligned to the participant’s input, and a second interview was arranged, and the same transcript was sent to the participant for verification.

### Trustworthiness

To ensure trustworthiness, the researchers followed Lincoln and Guba’s (1985) approach in conducting this qualitative study. The researchers ensured participants variability through establishing criteria for participation selection by being a foreign nurse educator. The interview guide was developed by all the authors. The researchers met frequently to read the transcripts repeatedly until they agreed on the meaning of the participant voices. An independent colleague, who is an expert in qualitative research, was also given five of the transcripts to read independently. The first author who conducted the interviews used reflexivity (Jootun et al. 2009), where she acknowledged her beliefs about being a foreigner working in another country and her views about foreigners.

### Ethical consideration

The study protocol was approved by the Higher Degrees Committee of the Department of Health Studies, University of South Africa (Reference number: HSHDC/866/2018). It was also approved by the Ministry of Health (Reference number: HPDME, 13/18/1). Written consent was obtained from all the participants before data collection. The principles of anonymity, written informed consent and voluntary participation were adhered to in this study. Participants were given detailed information about the study and why it was being conducted. Those who agreed to participate signed the consent form before the interviews. Their names and places of work were given in codes known to the researchers only. None of the participants was coerced to participate in the study.

## Findings

### Participants

A total of 13 participants were recruited for this study through a letter sent to each participant. This was followed by verbal consultations with the lead author. Four of the participants were women and nine were men. Nine of the participants were from Zimbabwe and four were from Zambia. Three of the women held a bachelor’s degree and one woman had a master’s degree. The nine men had master’s degrees and two of them were pursuing doctoral degrees through distance learning.

### Themes

The three themes that emerged from the findings are hostile behaviour, discrimination and inequitable application of procedures and processes. In the analysis of the theme ‘discrimination’, two subthemes emerged, namely, workplace injustice and individual injustice.

### Hostile behaviour

Hostile behaviours are a group of concepts that explain the harmful social interactions (Hutchinson & Jackson 2013). In the context of this article, the concept ‘hostile behaviours’ was defined according to participants’ experiences of mistrust, cynicism, negative beliefs about foreign nurse educators and attributions made to them.

All 13 participants reported hostile behaviour. Participants defined hostile behaviour as demeaning actions. Participants reported that they are subjected to insults and rude jokes by local colleagues regarding their foreignness. The following statements best captured these sentiments:

‘It is painful when someone laughs at your name and the way you talk and make derogatory remarks about it. It cuts deep! As if the insult is referring not just to you, but to your parents and clan as well … I found it totally disrespectful.’ (Participant 1, male, aged 45 years with 4 years working in Botswana)

‘It is not a joke to be called “mokwerekwere,” in reference to how I speak, because their dialect does not sound well to me, but I can never call them that. I found the name to be dehumanising.’ (Participant 4, female, aged 55 with 10 years working in Botswana)

‘One day, a colleague that … I thought we were friends … actually called me “Batola terata,” which means “those that jumped the fence.” I felt insulted. Reporting it to our principal did not help, as she said, “In Botswana it does not mean much … it is used to play.”’ (Participant 3, male, aged 46 with 7 years experience working in Botswana as a nurse educator)

### Discrimination

Discrimination was used from participants’ experiences as exclusion from other benefits because of their nationality. All 13 participants mentioned discrimination as a theme. This theme had two sub-themes, which were workplace injustice and individual injustice.

#### Workplace injustice

Workplace injustice is defined as unfair practices that create work disparities amongst employees and can result in negative health outcomes (Okechukwu et al. 2014). The authors further elaborate that workplace injustice targets those who are different from the more powerful groups. In this study, participants defined workplace injustice as exclusion from enjoying the benefits enjoyed by local nurse educators, and unfair terms and conditions of service.

Participants reported that they have experienced workplace injustice as they were not considered for promotion because they are foreigners. Apart from promotion, participants reported that they are excluded from government-assisted training to further their studies, as further studies are only for nationals. They also mentioned that they were recently told that if a local qualifies for a position, they will be considered ahead of a foreigner and the foreigner’s contract would be terminated, or they would be demoted. Participants’ sentiments in this regard are better captured by the following quotes:

‘Can you (researcher) imagine that we are assigned more committee work than they do; because they know, we are more committed than the locals are? However, when it comes to promotions, we are overlooked.’ (Participant 6, male, aged 42 years of age with 6 years working in Botswana)

‘For some of us (foreign nurse educators), who have been lucky to be promoted, we are told that, when a local with the same qualifications is found, we will be demoted or we have to go back home.’ (Participant 6, male, aged 42 years of age with 6 years working in Botswana)

#### Individual injustice

‘Individual injustice’ was also a sub-theme under discrimination. From the participants’ perspective, individual injustice refers to interpersonal behaviours that cause physical and psychological harm to themselves, originating from the supervisors, co-workers and at times the system they work under. Participants said they are regularly ridiculed by the locals. The following statements show how participants have experienced individual injustice:

‘Colleagues are fond of telling us, or talking to other colleagues in our presence, that a foreigner cannot come and take away their positions. It is time they go back, after all, we are not responsible for fights or their poor economy.’ (Participant 11, female, aged 52 with 12 years working in Botswana)

‘You work hard, because you want to buy your stay in Botswana. I take it easy, this is my country, our parents worked hard to realise this economy.’ (Participant 11, female, aged 52 with 12 years working in Botswana)

### Inequitable application of policies and processes

The last theme that emerged was that of ‘inequitable application of policies and processes’, which affects foreign nationals. Here participants believed that if indeed there are policies and processes that govern the employment of foreign nurse educators, all the institutions should apply them as they are, with no changes from one institution to another. Their arguments were based on the premise that all the institutions follow the same policies as mandated by the government of Botswana. It was a general belief that all government institutions should apply similar processes to all employees. However, the study participants reported that at one of the colleges, when the contracts of the foreign nationals are renewed, they retain their current position, even when there is a national who qualifies, and are not demoted, as is the case in other colleges. In other instances, participants have observed that recommendation letters for renewal of contracts of foreign nationals are done at least 3 months before expiry of contract, whereas in the case of locals it is done a week before. Participants see this as inequitable application of processes and policies. This view is supported by the following quotes from some participants:

‘We work for the same government, but policies and processes are not applied the same. For instance, in one institution my colleague was told that his contract would not be renewed and was told 6 months before time, whereas another colleague in a different institution was only told a month before his contract expired and that it would not be renewed.’ (Participant 8, female, aged 53 with 12 years working in Botswana)

‘We are used to contracts being automatically renewed. Since last year (2018) things began to change without us being notified. In other institutions foreign nurse educators are demoted once a national attains a master’s degree and in another institution they continue working in the same position until the end of contract where they would be told that it has not been renewed.’ (Participant 12, male, 56 years with 20 years working in Botswana)

## Discussion

The findings of this study revealed how foreign nurse educators experience incivility in Botswana. Incivility is an intimidating force that threatens the well-being of nurse educators in areas where it exists (Bambi et al. 2017). According to Abid et al. (2015), incivility includes explicit antisocial behaviour which is of greater intensity like aggression. In the context of this study, this is attributed to the participants’ foreignness. Muliira et al. (2018) stated that incivility flourishes in environments where the dominant group is allowed to continue their harassment with no consequence. The study further reports that some workers, including nurse educators, accept this act of incivility as a norm. Incivility from the findings of this article was experienced as hostile behaviour, discrimination and inequitable application of policies and procedures.

Hostile behaviour towards foreign nurse educators was experienced as a severe form of incivility. Makov et al. (2018) described hostile behaviour as behaviours that are intended to cause trouble to some individuals, and embrace dislike, aggression and anger. The hostility was also in the words and gestures demonstrated towards the foreign nurse educators. The participants in the study deemed the use of derogatory name-calling and attributing any wrongdoing to the foreign nurse educators as hostile behaviour. This finding is strongly supported by Greitemeyer and Sagioglou (2018); the study found that it is common for people of similar social networks to become hostile to those different from their own. Such hostility may result in both social and economic exclusion, as is the case with the current study.

Another finding of this study was that foreign nurse educators experience discrimination in the institutions where they are employed. Okechukwu et al. (2014) found that minority groups in organisations are most likely to suffer from workplace injustice (Yanar, Kosny & Smith 2018), and were of the view that vulnerable groups are more inclined to be exposed to unfair work practices – further supporting this assertion.

Selective application of policies and procedures was seen as another form of incivility. Lasiter, Marchiando and Marchiondo (2012) opined that policies that encourage incivility are the major cause of nurse educator shortages. The researcher argues that ‘discriminative policies result in failure to recruit foreign nationals who are capable and well qualified to fill the existing open vacancies for nurse educators’. Aragon (2018) supported this view and added that action on recruitment and retention should be done urgently, and all nurse educators regardless of race, colour, gender and nationality should be treated the same.

According to the Botswana Standard of Care of 2015, which is in line with the International Council of Nurses (ICN) code of Ethics, nurses must create an environment of caring and civility, and this applies to foreign nationals who come to Botswana as educators. According to Clark (2017), nurse educators have a unique role of being exemplary in creating an environment of civility in their workplace.

## Limitations

This study should be accepted within certain limitations. It employed a qualitative approach and the generalisability of the findings is not guaranteed. However, qualitative research does not aim at generalising findings but provides in-depth understanding of the phenomenon under study – in this case, the experience of foreign nurse educators in Botswana.

## Recommendations

In view of the expressed opinions by foreign nurses, the authors would like to recommend that authorities, particularly the policy administrators for all institutions, should put in place measures that would enable equal treatment amongst nurse educators. All academic staff should be offered equal opportunities in terms of teaching load, assignments, training and any other financial benefits. Furthermore, the academic administrators should also play a role in encouraging collegial interaction at work and outside the work environment.

## Conclusion

Foreign nurse educators working in Botswana experience incivility at the workplace. This research showed that foreign nurse educators experience hostile behaviour and discrimination in the workplace. Inequitable application of policies and processes was also a concern for them. On the basis of the current findings, the study strongly recommends application of equitable job opportunities to all employees, including foreign nurse educators who are employed to meet the shortage of nurse educators in Botswana. Nurse educator shortage will persist in Botswana, partly because of the experiences of incivility by foreign nurse educators, which in turn disrupts the overall teaching and learning of nursing students, demoralises faculty members and renders the profession of nurse educators unattractive (Palumbo 2018).

## References

[CIT0001] Abid G., Khan B., Rafiq Z. & Ahmed A., 2015, ‘Workplace incivility: Uncivil activities, antecedents, consequences; and level of incivility’, *Science International (Lahore)* 27(6), 6307–6312.

[CIT0002] Aragon S., 2018, Targeted Teacher Recruitment: What Is the Issue and Why Does It Matter?, Denver, Education Commission of the States, 80203-3460. http://www.ecs.org

[CIT0003] Bambi S., Guazzini A., De Felippis C., Lucchini A. & Rasero L., 2017, ‘Preventing workplace incivility, lateral violence and bullying between nurses. A narrative literature review’, *Acta bio-medica: Atenei Parmensis* 88(5S), 39–47. 10.23750/abm.v88i5-S.6838 PMC6357576 29189704

[CIT0004] Campbell E.K. & Oucho J.O., 2003, *Changing attitudes to immigration and refugee policy in Botswana*, Southern African Migration Project, Migration Policy Series 28, IDASA and Kingston, Cape Town.

[CIT0005] Clark C., 2017, *Creating and sustaining civility in nursing education*, 2nd edn., Sigma Theta Tau International, Indianapolis, Indiana.

[CIT0006] Creswell J.W., 2009, *Research design: Qualitative, quantitative, and mixed methods approaches*, 3rd edn., SAGE Publications, Los Angeles, CA.

[CIT0007] Greitemeyer T. & Sagioglou C., 2018, ‘The experience of deprivation: Does relative more than absolute status predict hostility?’, *British Journal of Social Psychology* 58(3), 515–533. https://doi.org/10.1111%2Fbjso.12288 10.1111/bjso.12288 PMC6618103 30375005

[CIT0008] Hutchinson M. & Jackson D., 2013, ‘Hostile clinician behaviours in the nursing work environment and implications for patient care: A mixed-methods systematic review’, *Biomedical Central Nursing* 12(1), Art. #25. 10.1186/1472-6955-12-25 PMC3851604 24094243

[CIT0009] Jootun D., McGhee G., & Marland G. R., 2009, ‘Reflexivity: Promoting rigour in qualitative research’, *Nursing standard (Royal College of Nursing (Great Britain): 1987)* 23(23), 42–46. 10.7748/ns2009.02.23.23.42.c6800 19263905

[CIT0010] Lasiter S., Marchiondo L. & Marchiando K., 2012, ‘Student narratives of faculty incivility’, *Nursing Outlook* 60(3), 121–126. 10.1016/j.outlook.2011.06.001 21840556

[CIT0011] Lincoln Y.S. & Guba E.G., 1985, *Naturalistic inquiry*, vol. 75, Sage, Thousand Oaks, CA.

[CIT0012] Ministry of Health, 2011, National health policy. ‘Towards a Healthier Botswana’, Bay Publishing (Pty) Ltd, Gaborone Printing and Publishing Company, viewed 20 October 2019, from http://www.moh.gov.bw/Publications/Policies/revised.

[CIT0013] Muliira J.K., Natarajan J. & Van der Colff J., 2017, ‘Nursing faculty academic incivility: perceptions of nursing students and faculty’, *Biomedical Central Medical Education* 17(1), Art. #253. 10.1186/s12909-017-1096-8 PMC5729510 29237443

[CIT0014] Okechukwu C.A., Souza K., Davis K.D. & De Castro A.B., 2014, ‘Discrimination, harassment, abuse, and bullying in the workplace: Contribution of workplace injustice to occupational health disparities’, *American Journal of Industrial Medicine* 57(5), 573–586. 10.1002/ajim.22221 23813664 PMC3884002

[CIT0015] Palumbo R., 2018, ‘Incivility in nursing education: An intervention’, *Nurse Education Today* 66, 143–148. 10.1016/j.nedt.2018.03.024 29704701

[CIT0016] Smith J.A. & Osborn M., 2015, ‘Interpretative phenomenological analysis as a useful methodology for research on the lived experience of pain’, *British Journal of Pain* 9(1), 41–42. 10.1177/2049463714541642 26516556 PMC4616994

[CIT0017] Spiri C., Brantley M. & McGuire J., 2017, ‘Incivility in the workplace: A study of nursing staff in the military health system’, *Journal of Nursing Education and Practice* 7(3), 40–46. 10.5430/jnep.v7n3p40

[CIT0018] Williamson M., 2018, ‘This is who we are: Promoting professional behaviours and civility in nursing education’, *Building Healthy Academic Community Journal* 2(1), 14–24. 10.18061/bhac.v2i1.6355

[CIT0019] Wright M. & Hill L.H., 2014, ‘Academic incivility amongst health science faculty’, *Adult Leaning* 26(1), 14–20. https://doi.org/10.1177%2F1045159514558410

[CIT0020] Yanar B., Kosny A. & Smith P., 2018, ‘Occupational health and safety vulnerability of recent immigrants and refugees’, *International Journal of Environmental Research and Public Health* 15(9), 2004 10.3390/ijerph15092004 PMC6165099 30223449

